# The Position of Nursing in Hospitals in Bristol

**Published:** 1956-04

**Authors:** Stephen C. Merivale

**Affiliations:** Secretary to the Board of Governors, United Bristol Hospitals


### THE POSITION OF NURSING IN HOSPITALS IN BRISTOL

BY

STEPHEN C. MERIVALE

*Secretary to the Board of Governors, United Bristol Hospitals*

It is only after considerable hesitation and, indeed, with some trepidation that a mere hospital administrator ventures to write upon such a subject as the position of nursing in hospitals. The author of this article was, however, emboldened to accept the Editor's invitation to do so for two compelling reasons. The first is the intrinsic importance of the subject. The second is that all those concerned in hospital management are necessarily also concerned in problems of nursing staffing and that the nursing staffing of hospitals is, in turn, inextricably entangled with the problems of nurse training. I myself believe that there is no conflict, in the widest context, between hospital management wishing to staff its hospitals with the best nurses in the largest possible numbers and the leaders of the nursing profession desirous of improving the standards of nurse training and the arrangements for carrying it out. This is because, in the last resort, and over the country as a whole, standards of staffing will both follow and reflect the standards of nurse-training, or, to put it bluntly, I think that the hospital service as a whole will get the nurses its deserves. Thus it behoves hospital management to give the closest attention to the views of the nursing profession on all matters of nurse-training, which views management can only ignore or overrule at considerable risk.

There is, however, no doubt that there are occasions when there does appear to be a conflict between the apparent best interests of the hospitals in getting nursing staff and the student nurses themselves in getting the best training. It is no doubt partly because of these occasions that the Nurses' Act of 1949 set up the bodies known as Area Nurse-Training Committees. These Committees are statutory bodies responsible to the General Nursing Council. They contain representatives of the hospital authorities such as the Regional Boards and Boards of Governors and, in this Region, the Secretary of the Regional Hospital Board is also the Secretary to the Area Nurse-Training Committee. But it is the Area Nurse-Training Committee and not the Regional Board, Board of Governors or Hospital Management Committee which both

(*a*) approves arrangements made by hospital authorities for the training of nurses for all the various nursing registers and

(*b*) provides the necessary finance for such training, i.e., the salaries of nurse tutors, the fees of lecturers, the cost of equipment, &c.

The money provided under (*b*) above is separately voted by Parliament and passed to Area Nurse-Training Committees through the General Nursing Council. The object of separating this from the ordinary Health Service vote is presumably to ensure that in times of retrenchment and financial stringency, hospital authorities cannot look to nurse-training as one of their activities upon which it would be convenient to save money.

Such, then is part of the background—the administrative arrangements in the Service for dealing with nurse-training. The other, or larger half of the background is the training itself. Neither the profession, nor the Health Service has been quick to seize upon the recommendations of the pre-war Working Party on the Recruitment and Training of Nurses. There has, as yet, been no attempt to merge separate registers maintained by the General Nursing Council for general-trained nurses, mental-trained nurses, mental-defective-trained nurses, sick children's trained nurses or fever-trained nurses. These five certificates have to be obtained separately in different training schools. There are also separate certificates awarded for orthopaedic and ophthalmic nursing. Finally an interesting and fairly recent development is the S.E.A.N. or State-Enrolled Assistant Nurse.

There are in Bristol and its immediate environs four Hospital Groups containing general training schools for nurses: Southmead, Cossham/Frenchay, the Homoeopathic and the Bristol Royal Hospital. There are two hospitals, Bristol Mental and Barrow Gurney, each with a complete school for mental training. There are four training schools for nursing the mentally defective, at Hortham, Brentry, Stoke Park and Purdown. For assistant nurses there are three training schools, Stapleton Hospital, Snowdon Road with the Walker Dunbar Hospital, and Berkeley and Thornbury Hospitals jointly. In addition nurses can train for the fever register at Ham Green, for the sick children's register at the Royal Hospital for Sick Children, and for the orthopaedic and ophthalmic certificates respectively at Winford Orthopaedic Hospital and at the Bristol Eye Hospital.

And now let us look at how these arrangements are working out in practice purely from the point of view of hospital management. First of all recruitment in the Bristol area is, generally speaking, healthy. The only rule that it seems to me safe to rely upon in the matter of recruitment of nurses is that there appear to be no rules! This statement is, of course, something of an exaggeration. The great London Teaching Hospitals enjoy and will presumably continue to enjoy, a superfluity of choice of candidates. Indeed, it is the normal thing for them to have 1,000 candidates for 200 places. We in the provinces are perhaps in one sense fortunate in that we can never expect to have such heavy responsibilities thrust upon us. We do, however, not infrequently have to exercise a choice and attempt to direct candidates aright. In the eighteenth century the Bristol Royal Infirmary, in need of a new matron, advertised for " a discreet and managing woman " and I believe the truth to be that in the provinces good matrons with powers both of management and of discretion, with a good hospital offering a good nursing training can recruit the students, not necessarily in the numbers required, but certainly nearly up to the establishment approved. It is, however, always necessary, in fairness to those whose hard task it is to recruit nurses, to remember the harsh limitations imposed by our national circumstances upon their work. There never have been and it is not likely that there ever will be, in any given year, enough girls born eighteen years previously of General Certificate of Education standard to "man" all the professions for women. It is, alas, arithmetically true that if nursing successfully claimed the lot there would not only be none left for teaching and the other professions but there would still be hospital beds unstaffed by nurses. This makes it all the more important to make the best use of all the students who do come forward for training and one of the most encouraging aspects of this is the enormous growth since 1948 of the assistant nurse training schools. In the South-West Region as a whole in 1948 there were 55 students entering 9 hospitals and in 1954 there were 266 entrants to 43 hospitals offering training to assistant nurses. This is a remarkable achievement not sufficiently generally known.

Other figures of interest are that the four general training Schools in Bristol have a combined establishment for 770 students and, at the time of writing, there are 586 nurses in training. With a rate of " wastage" much lower than the national figure this represents a very substantial increase in numbers since 1948 and the figures are still going up, a position which reflects creditably on those concerned. The position is not so favourable as regards recruitment to either mental, mental-deficiency or fever nursing. The first two, however, are improving and the layman may perhaps be forgiven for thinking that the position as regards fever-training is overdue for revision. Chemotherapy has completely altered the traditional picture of the fever hospital of twenty years ago, which we shall certainly not see again.

To return once more to the recommendations of the Working Party Report (which repays another reading) both the doctor and the simple layman may perhaps be forgiven if they exclaim in astonishment that these matters, nationalized some six years past, have not already been rationalized too. Even allowing for "the leaden foot" of a nationalized bureaucracy it might be thought that before now someone could have improved upon arrangements which involved in an area the size of Bristol 16 different training schools for nurses teaching their students for no less than 5 different registers and 2 separate certificates. There is some force in such an argument but it is also as well to remember that there are considerations on the other side. These are:

(i) Nothing can be done on a large scale towards "rationalizing" nurse-training until a national lead is given by the General Nursing Council which has so far made no move in the matter.

(ii) A good deal has in fact been done in Bristol, as elsewhere, in the admittedly limited way that is all that the present regulations of the General Nursing Council allow, to give nurses the opportunity of a "comprehensive" training. Thus student nurses in the general hospitals go to Ham Green for three months' fever experience in their second year and this counts in their time programme if they later decide to do a fever training. In the same way nurses can choose to do in four years a complete training for both the General and Sick Children's Register between the Bristol Royal and Bristol Royal for Sick Children Hospitals. There are links between the ophthalmic and orthopaedic certificates and general training schools in much the same way. Other " comprehensive" arrangements are on the way between general students and the geriatric and mental hospitals. These wait only upon a further, confidently anticipated, improvement in recruitment. Finally much has been done in the sharing of facilities in preliminary training schools to concentrate the work and economize in the time of tutors and lecturers to nurses.

(iii) There is really a good deal to be said in favour of most of the present training schools and at the least one may be certain that it would be no substitute to recruit nurses to one " Bristol Training School". I cannot envisage ever building up an "esprit de Bristol" which would effectively substitute for the *esprit de corps* of the existing training schools with their individual traditions, both old and new, their special peculiarities and their own intimate connexions both geographical and otherwise.

(iv) Finally it must be faced that the different training schools can deal effectively with students of different educational attainments. A girl who might not make the grade in School " A " will successfully train in "B" and become an excellent practical nurse.

In conclusion I hope that your Editor will feel able to devote space to a further article on the situation and developments recounted above written from the point of view of nurse-training not from that of hospital management. But that article will have to be written by a nurse.

